# Hybrid repair of an aortocaval fistula and inferior vena cava external compression caused by an inflammatory aortoiliac aneurysm: a case study

**DOI:** 10.1186/2193-1801-3-476

**Published:** 2014-08-27

**Authors:** Tatsuo Banno, Hokuto Akamatsu, Ryota Hanaoka, Hiroshi Toyama, Ryoichi Kato

**Affiliations:** Department of Radiology, Fujita Health University School of Medicine, 1-98, Dengakugakubo, Kutsukake, Toyoake, Aichi, 470-1192 Japan

**Keywords:** Aortocaval fistula (ACF), Abdominal aortic aneurysm (AAA), Stent graft, Iliofemoral bypass, Inferior vena cava (IVC)

## Abstract

**Introduction:**

We report a case of aortocaval fistula successfully treated by hybrid operation.

**Case description:**

A 73-year-old female suffering from malignant lymphoma and painful leg edema was transferred to our institution. Computed tomography revealed an aortoiliac aneurysm. The inferior vena cava was compressed by displacement of the abdominal aortic aneurysm. The bilateral internal iliac and ovarian veins were markedly dilated. Diagnosis was an aortoiliac aneurysm with aortocaval fistula. The treatment options were open surgery or an intervention with bypass surgery. Because of narrow iliac access for a bifurcated stent graft, aorto-uni stentgraft treatment followed by bypass surgery was finally decided. Following stent graft insertion and iliofemoral artery bypass, the aneurysms and fistula were successfully excluded without endoleaks. To treat the inferior vena cava compression, the kissing technique was used to place bare metallic stents across the bilateral common iliac veins and inferior vena cava, which improved the clinical symptoms.

**Discussion and Evaluation:**

In this aortocaval fistula caused by AAA, a minimally invasive treatment of stentgraft and bypass surgery with venous flow recovery was chosen as a hybrid treatment. Intravascular intervention was the most suitable in this situation. Bare stent placement for venous occlusion was also effective for revascularization of vena cava flow.

**Conclusion:**

Recent advances in endovascular devices, including stent grafts and bare metallic stents, will be helpful for effective noninvasive treatment for aortocaval fistula circulation.

**Electronic supplementary material:**

The online version of this article (doi:10.1186/2193-1801-3-476) contains supplementary material, which is available to authorized users.

## Background

Aortocaval fistula (ACF) is a comparatively rare pathology with a high operative mortality rate of 21%–55% (Alexander and Imbembo 1989). Rapid deterioration in surgical invasiveness and large perioperative changes in the hemodynamic status are believed to contribute to this high mortality (Ghilardi et al. 1993). Measures such as early diagnosis and prompt surgical intervention are therefore important in patients with ACF. Symptomatic ACF has been repaired through open surgery. These procedures can be technically challenging, with high operative mortality rates approaching 30% (Mitchell et al. 2009). Recently, reports concerning endovascular treatment employing endoprosthesis have been increasing (Godart et al. 2005; Vetrhus et al. 2005; Melas et al. 2011a, [b]).

We report a case of ACF successfully treated using a hybrid combination of stent graft insertion and kissing stent technique for inferior vena cava (IVC) compression in a patient with an abdominal aortic aneurysm (AAA).

## Case description

A 73-year-old female with a history of thoracic surgery had been diagnosed with malignant lymphoma. During treatment, an aneurysm of the abdominal aorta and right common iliac artery (CIA) was found on a computed tomography (CT) scan. Because CT performed in the previous month showed a normal aorta, the aneurysm was believed to be an inflammatory process. The patient was transferred to our hospital for treatment of the aneurysm. On admission, she presented with painful right leg edema that had begun 1 month earlier. Laboratory findings on admission were positive for hepatitis C virus antibody, pancytopenia, liver dysfunction, and heart failure. There was no laboratory data suggesting inflammation.

### Radiographic findings and treatment indications on admission

**Figure 1:**
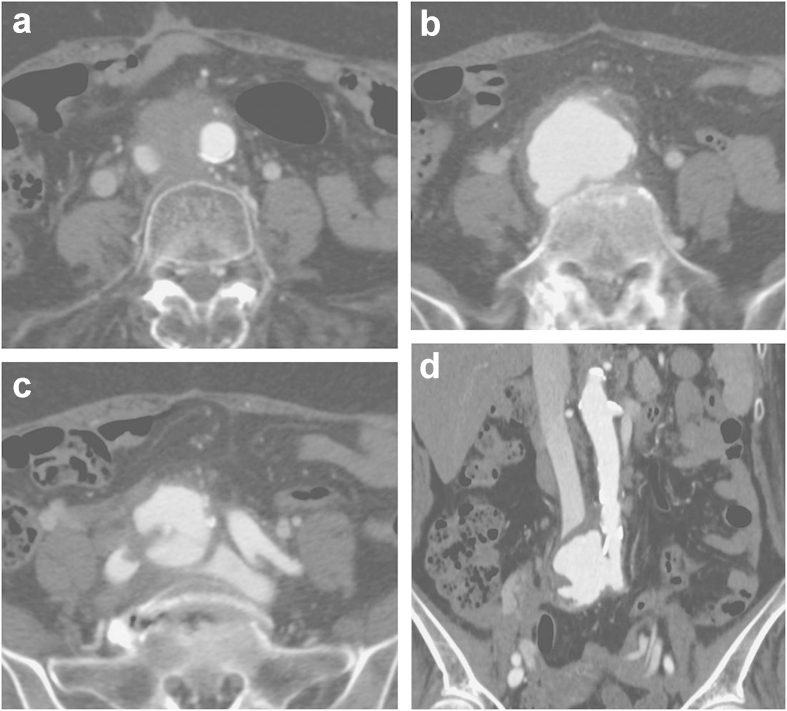
**Contrast-enhanced CT imaging. a)** Contrast-enhanced CT at the level of the lower abdominal aorta. A nonenhancing mass is recognized in the aortocaval space. The IVC is compressed and narrowed. **b)** Contrast-enhanced CT at the level of the terminal aorta. A large aneurysm with an irregular shape is recognized. **c)** Contrast-enhanced CT at the level of the CIA. The aneurysm and normal left CIA are visualized. Early venous filling of the left CIV and filling defect of the right CIV are recognized. **d)** Coronal multiplanar reconstruction contrast-enhanced CT image. An aneurysm arising at the terminal aorta and compressed IVC are recognized.

**Figure 2:**
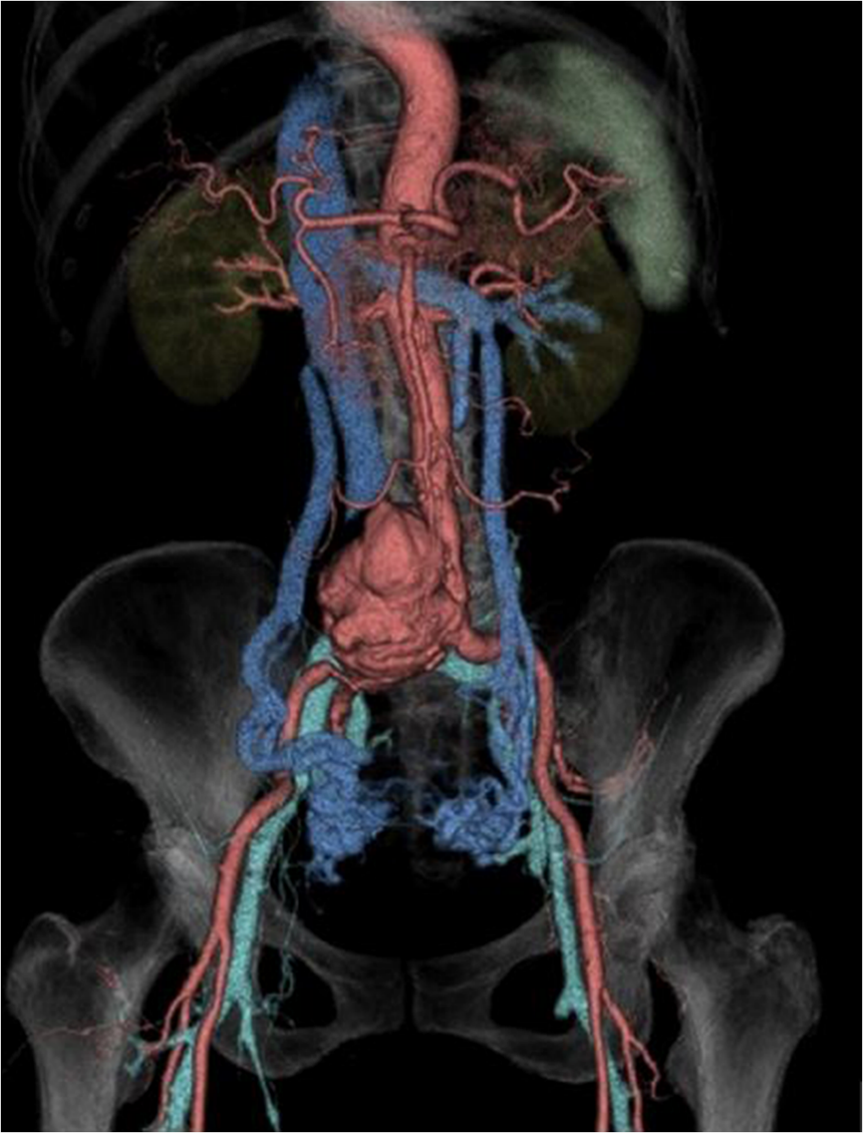
**Preoperative 3D volume rendering image in the anterior view.** A large aneurysm from the lower part of the abdominal aorta to the right CIA is observed. There is stenosis of the IVC due to displacement of AAA. The fistulous venous return to the IVC runs via the peripheral dilated veins in the bilateral internal iliac veins, dilated left and right ovarian veins, and left renal vein.

Contrast-enhanced CT showed an aneurysm at the level of the terminal aorta, IVC compression, and early venous filling into the iliac veins (Figure 1a–1d). CT angiography (CTA) revealed AAA and right CIA aneurysm (Figure 2). The IVC was compressed at the lower portion near the confluence of the bilateral common iliac veins (CIVs) by displacement of AAA. The bilateral internal iliac veins and bilateral ovarian veins were dilated, which was suggestive of ACF. The general condition of the patient was stable, and the aneurysm was suitable for stent grafting. Because of the narrow diameter of the abdominal aorta (14 mm) and external iliac artery, a bifurcated stent graft was not suitable for insertion. After informed consent was obtained from the patient, we decided to insert a straight stent graft between the abdominal aorta and left CIA after coil embolization of the right internal iliac arteries. Iliofemoral artery bypass from the left external iliac artery to the right femoral artery was followed after stent graft insertion.

### Treatment method

**Figure 3:**
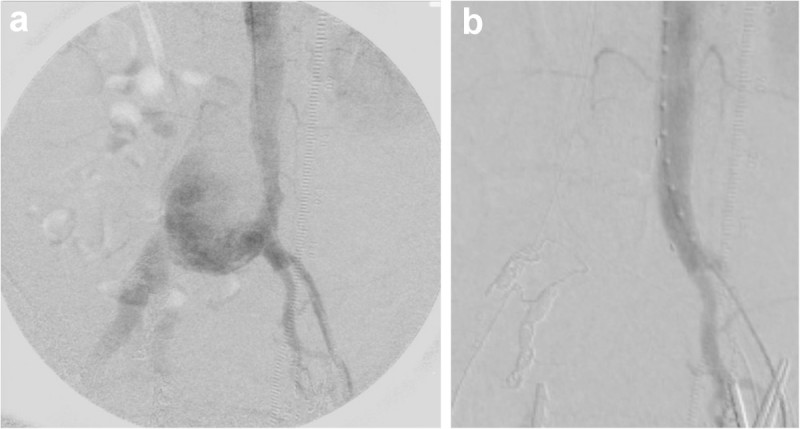
**Angiographic imaging. a)** The initial abdominal aortic angiogram. A large aneurysm arising from the lower abdominal aorta to the right CIA is observed. There is also early venous filling in the right CIV. **b)** An angiogram after stent graft placement. The aneurysm is excluded. No endoleaks are observed. Early venous filling of the right CIV has disappeared.

The bilateral femoral arteries were exposed and looped under general anesthesia. A 4-Fr sheath (Radifocus Introducer II; Terumo, Tokyo Japan) was then inserted into the right cubital vein, and an intravenous route was established. Aortic angiography revealed a terminal aortic aneurysm extending to the right CIA. The adjusted IVC was obliterated because of external compression caused by the aneurysmal sac. Early venous filling of the internal iliac veins and ovarian veins was also visualized (Figure 3a). These findings suggested that ACF was caused by the rupture of the right CIA aneurysm with IVC compression due to displacement of the aneurysmal sac and development of collateral circulation in the pelvic venous system.

From the right transfemoral artery, the right internal and external iliac arteries were embolized by using detachable micro coils to prevent endoleaks. A through-and-through technique was used from the right cubital vein through the right femoral vein. After making a small incision at the left iliac artery, forceps were used to clamp the distal portion. A 14-Fr sheath (Ultimum™ EV Hemostasis; St. Jude Medical, USA) was inserted into the abdominal aorta along a preloaded stiff guide wire (Amplatz Ultrastiff guidewire; Cook Medical, USA). A stent graft (Excluder Leg®, 16 mm–8 cm; W.L. Gore, AZ, USA) was inserted and deployed at the portion between the abdominal aorta just below the inferior mesenteric artery orifice and the distal portion of the left CIA. Then, a 27-mm Equalizer (Equalizer™ Occlusion Balloon Catheter, Boston Sientific,USA) was used to apply balloon touch-ups. In addition, percutaneous transluminal angioplasty balloon (XXL, 16 mm × 2 cm; Boston Scientific, USA) dilatation was applied at the proximal and distal portions of the stent graft. Aortic angiography showed no endoleaks after stent graft insertion (Figure 3b).

**Figure 4:**
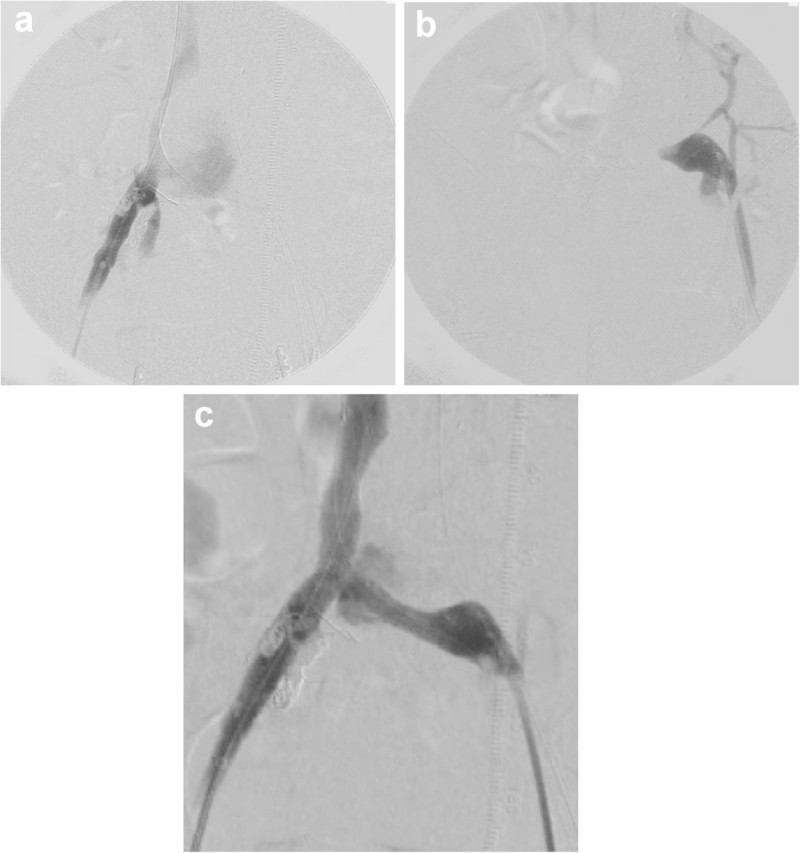
**Venographic imaging. a)** Venography from the right femoral vein immediately after stent graft insertion. The contrast agent has filled the excluded aneurysmal sac. The IVC is visualized but compression is still present. **b)** Venography from the right femoral vein immediately after stent graft insertion. The left CIV is visualized; however, it is occluded. **c)** Bilateral femoral vein angiogram after kissing technique stent insertion. Severe stenosis in the IVC has improved, and caval venous flow has recovered after insertion of bare metallic stents. Minimal contrast agent filling of the aneurysmal sac is observed.

Right femoral vein angiography confirmed the entry of contrast agent into the aortic aneurysm and narrowing of the IVC (Figure 4a). Left femoral vein angiography showed obliteration of the left CIV (Figure 4b). Bare metallic stents of 12 mm × 10 cm and 10 mm × 10 cm (Luminexx®; Bard PV, AZ, USA) were then inserted at the same site from the right and left femoral veins, respectively, using the kissing method. After insertion of the bare metallic stents, venography of the bilateral femoral veins showed good flow from the femoral vein to the IVC (Figure 4c). Following longitudinal incision to the left external iliac artery, iliofemoral artery bypass using an 8-mm ePTFE graft with rings (W.L. Gore, AZ, USA) was completed.

### Postoperative radiological findings and clinical course

**Figure 5:**
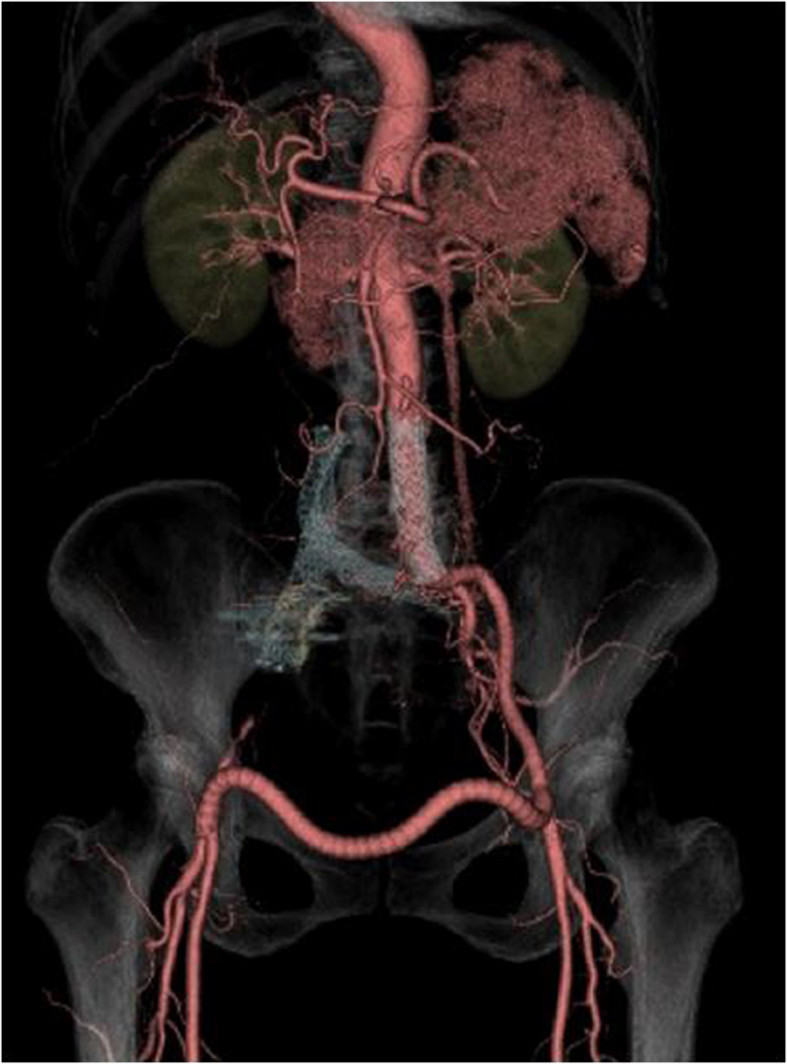
**Postoperative 3D CTA, anterior view.** There is no endoleak in the stent graft. The fistulous flow has also disappeared. The left external iliac artery to the right common femoral artery (I–F) bypass flow shows good patency.

After treatment, the aneurysm was completely excluded. Early venous filling also disappeared, and good venous returns were observed. Iliofemoral bypass showed normal flow into the bilateral femoral arteries (Figure 5). Clinically, the patient’s leg pain and edema disappeared. Cardiac symptoms and laboratory data, including liver dysfunction data, were reviewed. There was no evidence of recurrence at 3.5-month follow-up CT.

## Discussion and evaluation

Ruptured AAA can perforate neighboring structures such as the intestinal tract, IVC, and renal veins, but ACF is comparatively rare and accounts for only a small percentage of all ruptured AAAs [3%–4% (Alexander and Imbembo 1989; Baker et al. 1972) or 2%–4% (Brewster et al. 1991)]. Symptoms of ACF vary depending on the duration and rate of blood flow through the fistula and can include abdominal pain, back pain, high output, bilateral leg pain and edema, kidney dysfunction, hematuria, and liver dysfunction. The classic triad of symptoms consists of back pain, palpable AAA, and machinery murmur (Farid and Sullivan 1996). Another noteworthy feature of this pathology is the high operative mortality rate of 30%–36.4% (Ghilardi et al. 1994; Davidovic et al. 2002). This may be attributable to the deterioration in surgical tolerance of patients with ACF.

ACF causes high-output cardiac failure, which progresses relatively quickly to advanced cardiac failure. Renal failure and liver failure can also arise through a decrease in renal blood flow due to elevated renal vein pressure and reduced renal artery pressure and a decrease in liver blood flow due to elevated hepatic vein pressure. The large changes in the hemodynamic status before and after surgery may also influence operative mortality rates (Ghilardi et al. 1993). Measures such as early diagnosis and prompt surgical intervention are important in patients with ACF. Multiphasic contrast CT and color Doppler ultrasonography should be useful to establish early diagnosis. Symptomatic ACF has traditionally been repaired using open surgery. The procedures can be technically challenging, with significant intraoperative blood loss and high operative mortality rates approaching 30% (Mitchell et al. 2009).

Recently, reports concerning endovascular treatment employing endoprostheses have been increasing (Godart et al. 2005; Vetrhus et al. 2005; Melas et al. 2011a,[b]). In the present case, clinical symptoms had not deteriorated very much because of the fistulous flow behavior. Bulging of the aneurysmal sac had compressed the IVC, which resulted in stenosis of the vena cava. The fistulous flow did not return directly to the proximal segment of the IVC. Instead, the blood flow eventually returned to the IVC via a collateral route from the bilateral ovarian veins to the renal veins. Thus, the pressure increase in the IVC proximal from the fistula was probably suppressed to a certain extent, and heart failure and renal dysfunction did not occur. Significant blood flow from the distal IVC to the iliac veins caused painful leg edema.

Concerning the treatment of the aneurysms, we selected a straight stent graft from the abdominal aorta to the left CIA instead of a bifurcated stent graft because of the narrow diameter of the abdominal aorta and use of the external iliac artery as an access route. Usually, the main bifurcated EVAR device needs an 18-Fr or 20-Fr outer diameter sheath for deploying the stent graft. In these cases involving a narrow aorta, the leg component of EVAR devices is useful for access and fitting of the aortic caliber.

Treatment options for venous occlusion, particularly in the vena cava or common iliac veins (CIVs), are controversial. In ACF, decompression of the aneurysmal sac using a stent graft may release the venous flow. However, thromboembolic risk, including pulmonary embolism, is inevitable. To reduce a massive thromboembolism, a metallic stent in the venous system was believed to be effective for maintaining the flow. In our treatment of IVC compression at the confluence of both CIVs, we used the kissing technique to insert bare metallic stents into both femoral veins. Vena cava compression usually results in bilateral CIV occlusions. In this occlusive case, insertion of a bare metallic stent via the kissing technique demonstrated great efficacy for flow recovery in the venous channels.

In ACF cases, the treatment options include open surgery, interventional treatment, and a hybrid treatment of intervention and open surgery. In open surgery, both IVC stenosis and aneurysm are repaired. However, the invasiveness of the procedure may result in an overall deterioration of the patient’s condition. Because the patient’s status was determined to be relatively stable, we selected hybrid treatment. An additional factor in our decision was IVC compression caused by the aneurysm that required placement of bare metallic stents for revascularization from the occlusive vena cava. Therefore, we reasoned that intravascular intervention was the most suitable indication in such a situation. In this case, stent placement using the kissing technique in both CIVs up to the IVC was effective for revascularization of vena cava flow that caused symptomatic lower leg edema.

## Conclusion

In summary, we reported a case of ACF that was successfully treated by stent graft insertion with additional surgical iliofemoral bypass. Recent advances in endovascular devices, including stent grafts and bare metallic stents, will be helpful for effective noninvasive treatment of ACF circulation.

## Consent

Written informed consent was obtained from the patient’s husband for publication of this case report and any accompanying images.

## Authors’ original submitted files for images

Below are the links to the authors’ original submitted files for images. Authors’ original file for figure 1 Authors’ original file for figure 2 Authors’ original file for figure 3 Authors’ original file for figure 4 Authors’ original file for figure 5 Authors’ original file for figure 6 Authors’ original file for figure 7 Authors’ original file for figure 8 Authors’ original file for figure 9 Authors’ original file for figure 10 Authors’ original file for figure 11
