# Supplementary figures and images for: Hybrid repair of an aortocaval fistula and inferior vena cava external compression caused by an inflammatory aortoiliac aneurysm: a case study
Source: Springerplus. 2014 Aug 27;3:476. doi: 10.1186/2193-1801-3-476 (PMC4797187; doi:10.1186/2193-1801-3-476)

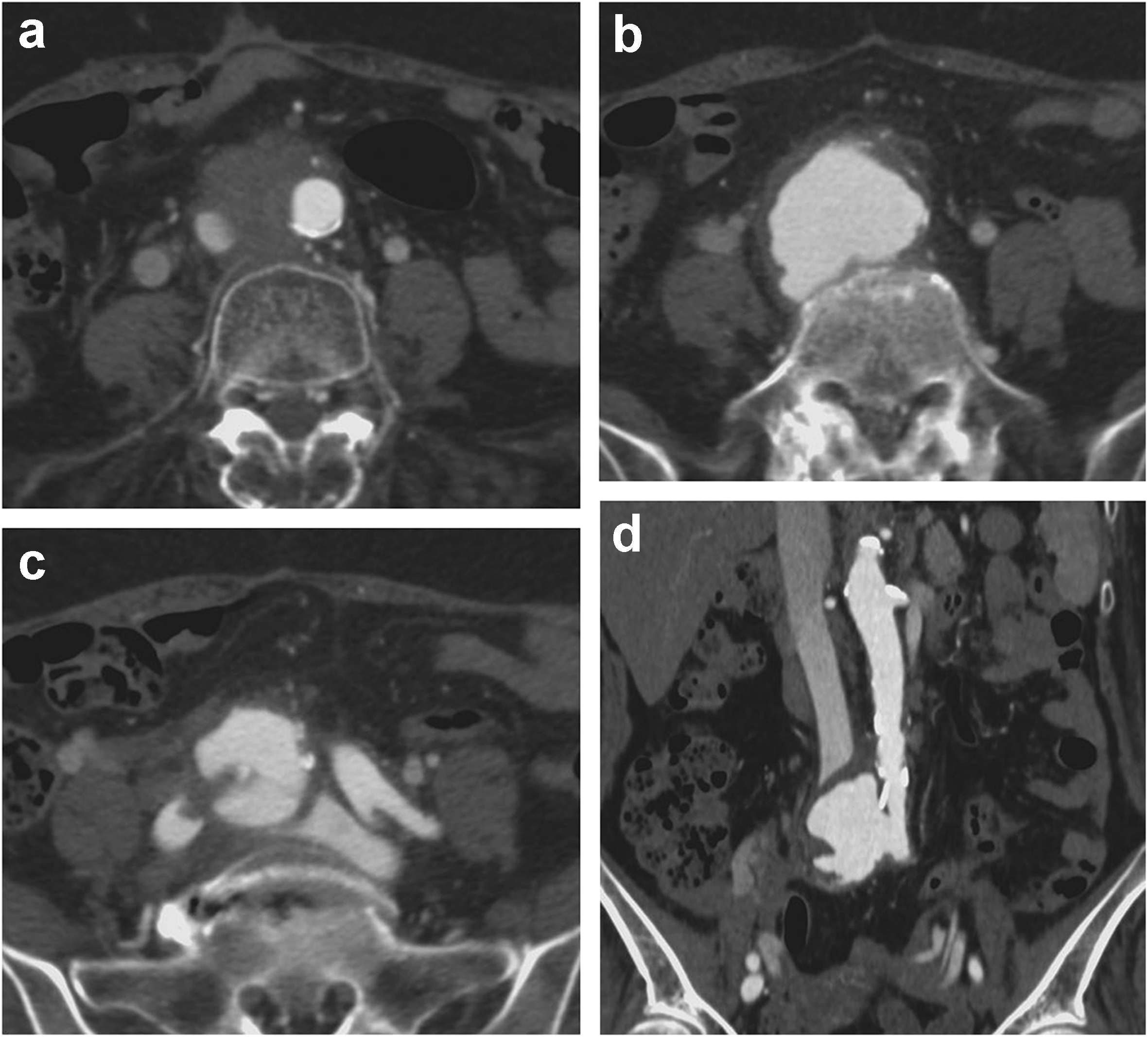

Supplement: Supplementary file 1 — Authors’ original file for figure 1 [file 40064_2014_1218_MOESM1_ESM.tif]

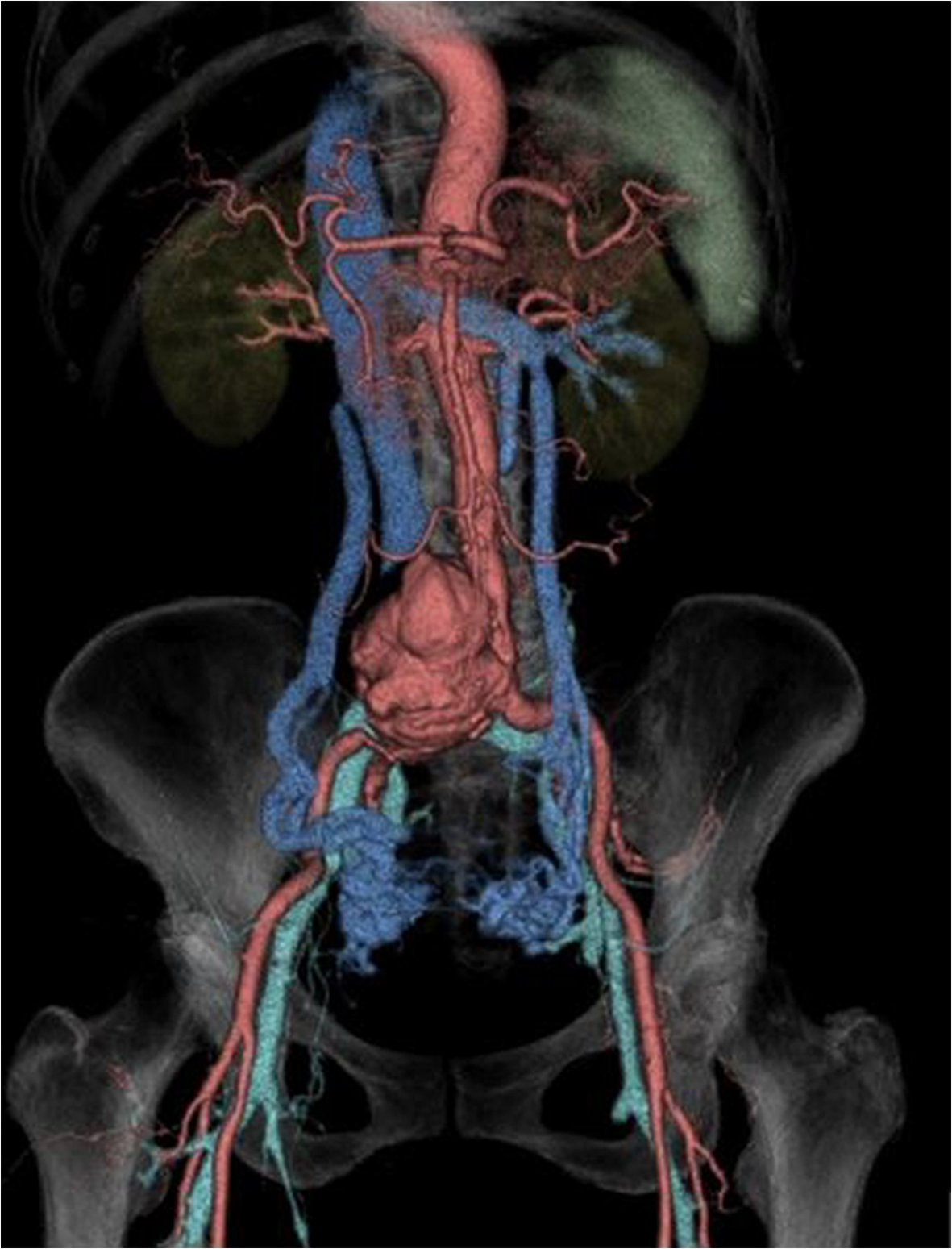

Supplement: Supplementary file 2 — Authors’ original file for figure 2 [file 40064_2014_1218_MOESM2_ESM.tif]

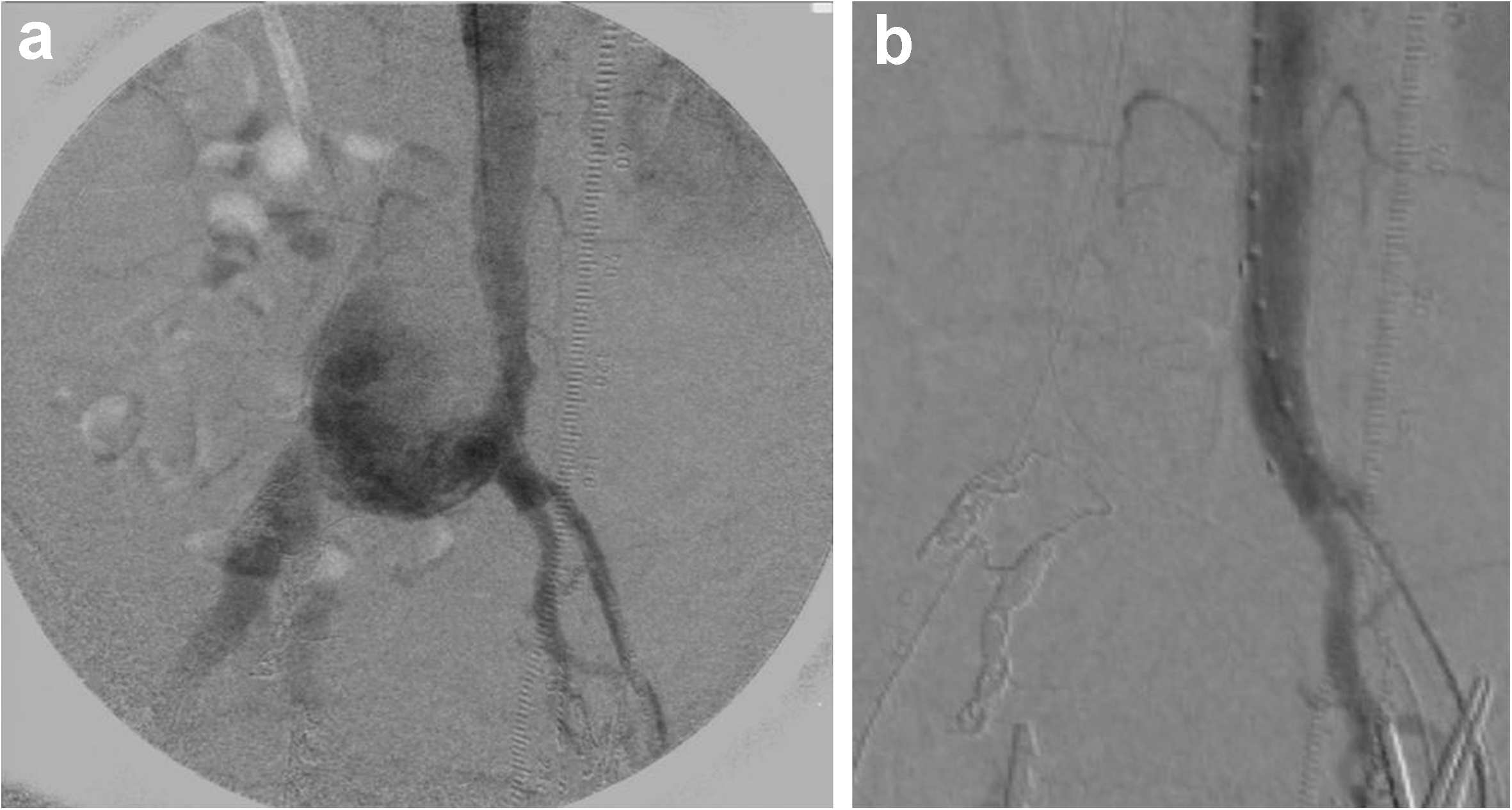

Supplement: Supplementary file 3 — Authors’ original file for figure 3 [file 40064_2014_1218_MOESM3_ESM.tif]

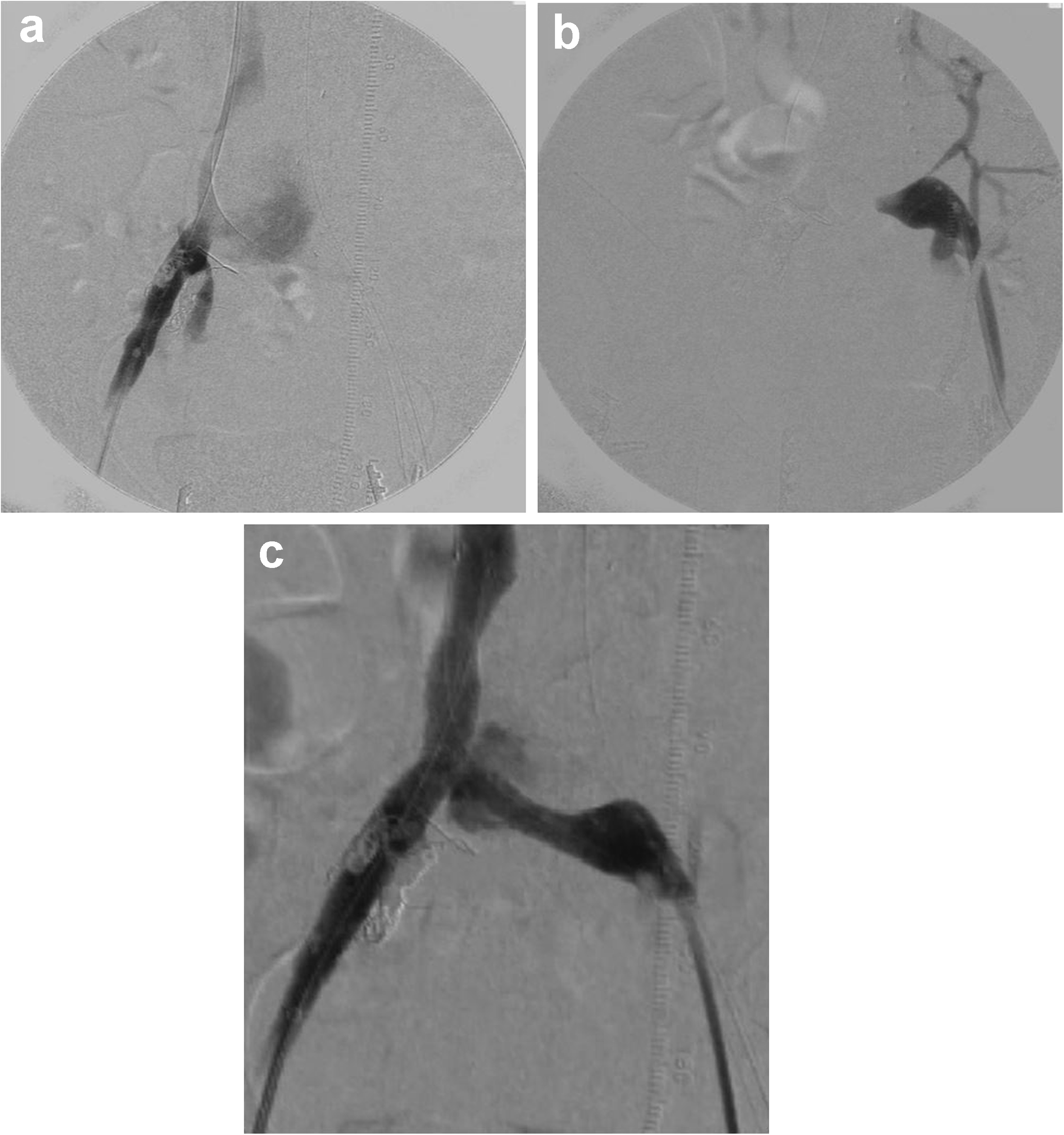

Supplement: Supplementary file 4 — Authors’ original file for figure 4 [file 40064_2014_1218_MOESM4_ESM.tif]

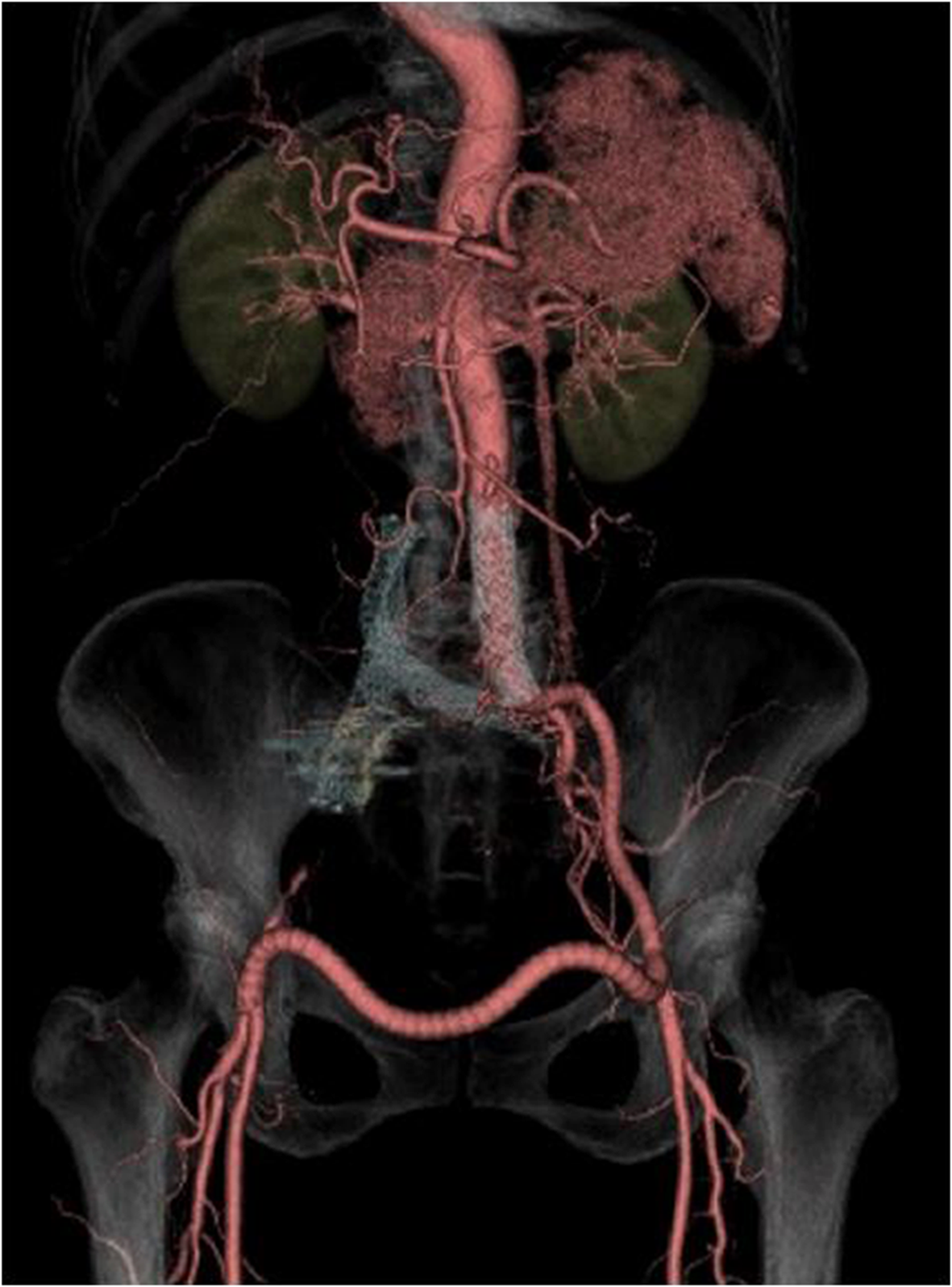

Supplement: Supplementary file 5 — Authors’ original file for figure 5 [file 40064_2014_1218_MOESM5_ESM.tif]

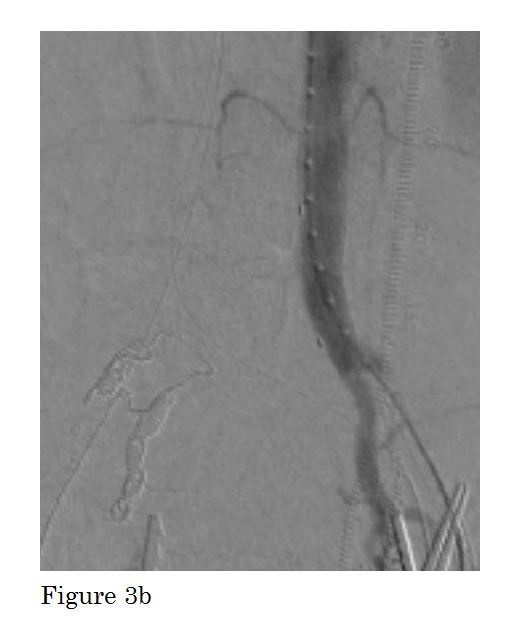

Supplement: Supplementary file 7 — Authors’ original file for figure 7 [file 40064_2014_1218_MOESM7_ESM.jpeg]

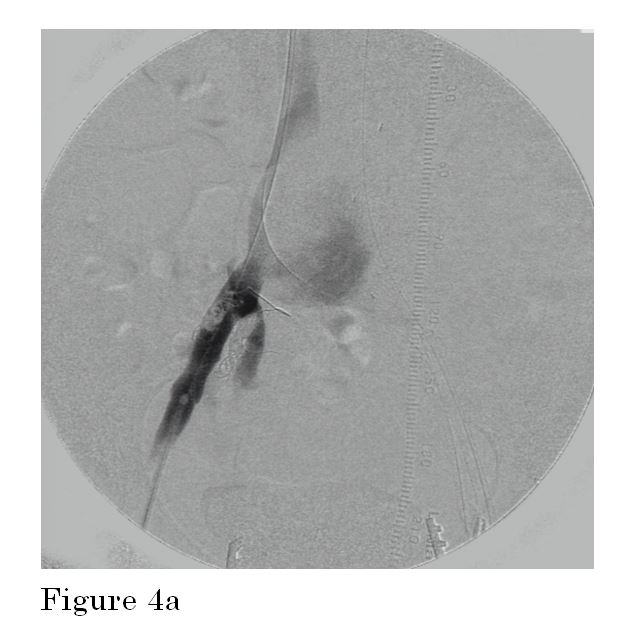

Supplement: Supplementary file 8 — Authors’ original file for figure 8 [file 40064_2014_1218_MOESM8_ESM.jpeg]

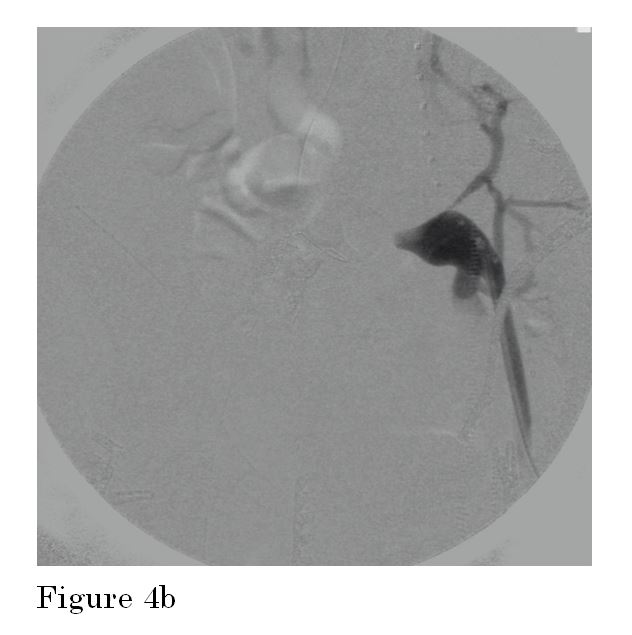

Supplement: Supplementary file 9 — Authors’ original file for figure 9 [file 40064_2014_1218_MOESM9_ESM.jpeg]

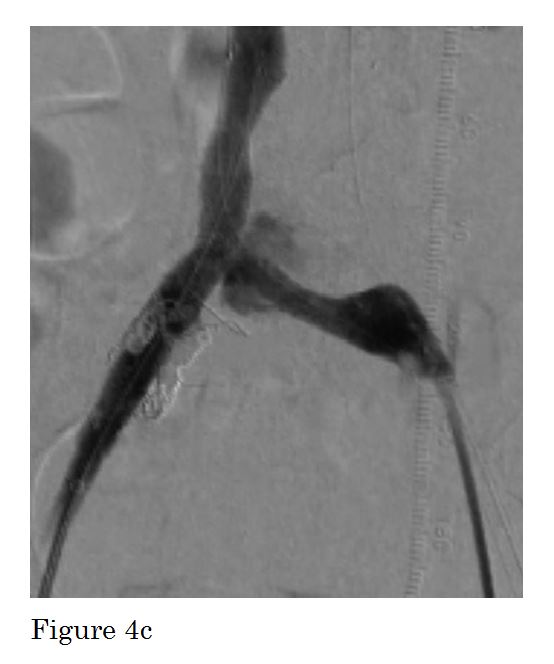

Supplement: Supplementary file 10 — Authors’ original file for figure 10 [file 40064_2014_1218_MOESM10_ESM.jpeg]

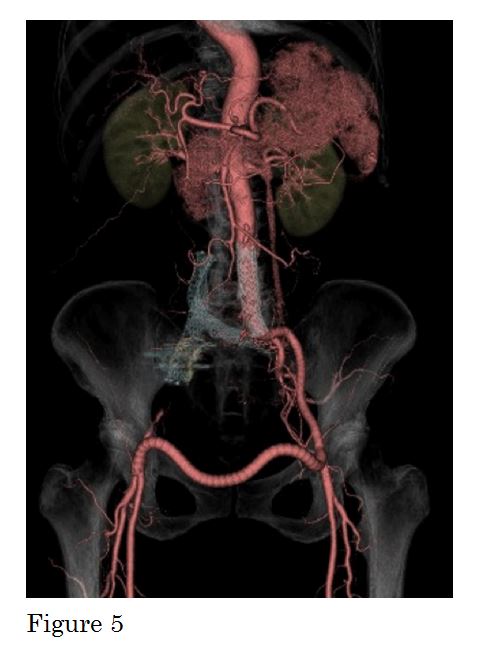

Supplement: Supplementary file 11 — Authors’ original file for figure 11 [file 40064_2014_1218_MOESM11_ESM.jpeg]
